# Toward improving reproducibility in neuroimaging deep learning studies

**DOI:** 10.3389/fnins.2024.1509358

**Published:** 2024-12-02

**Authors:** Federico Del Pup, Manfredo Atzori

**Affiliations:** ^1^Department of Information Engineering, University of Padua, Padua, Italy; ^2^Department of Neuroscience, University of Padua, Padua, Italy; ^3^Padova Neuroscience Center, University of Padua, Padua, Italy; ^4^Information Systems Institute, University of Applied Sciences Western Switzerland (HES-SO Valais), Sierre, Switzerland

**Keywords:** deep learning, reproducibility, replicability, repeatability, open source, BIDS

## 1 Introduction

After more than a decade since the Imagenet breakthrough (Krizhevsky et al., 2012), there is no doubt that Deep Learning (DL) has established itself as a powerful resource whose limits and risks remain difficult to assess (Bengio et al., 2024). This success, originated from the ability of deep neural networks to create representations of complex data with multiple levels of abstraction (LeCun et al., 2015), has inevitably attracted researchers from different domains, including multidisciplinary ones.

In computational neuroscience (Trappenberg, 2009), deep learning has offered novel insights into the functionalities of the brain, demonstrating a remarkable ability to exploit the intrinsic multimodality of the field (Saxe et al., 2021). This is also reflected in the increasing volume of publications encompassing diverse data types such as electroencephalographam (EEG), magnetoencephalographam (MEG), structural (MRI) and functional magnetic resonance imaging (fMRI) (Zhu et al., 2019; Zhang et al., 2021). Nevertheless, the potential of deep learning in neuroimaging data analysis is countered by several critical issues (Miotto et al., 2018), including the scarcity of large open datasets, the poor generalizability of DL models, their lack of interpretability, and the poor reproducibility of results, which is discussed in this work.

According to National Academies of Sciences, Engineering, and Medicine et al. (2019), reproducibility is defined as the ability to “obtain consistent results using the same input data; computational steps, methods, and code; and conditions of analysis.” This definition differs from that of replicability, commonly mistaken as a synonym, which is instead defined as the ability to “obtain consistent results across studies aimed at answering the same scientific question, each of which has obtained its own data.” From the above definitions, it is possible to derive how reproducibility solely depends on how authors facilitate the emulation of the same computational environment. Improving reproducibility, especially in a deep learning scenario, is therefore crucial for ensuring methodological robustness and result trustworthiness. However, when analyzing neuroimaging deep learning studies, several researchers have expressed concerns on how authors rarely report the key elements that make a study reproducible (Ciobanu-Caraus et al., 2024; Colliot et al., 2023). A recent review of DL applications for medical image segmentation found that only 9% of the selected studies were reproducible (Renard et al., 2020), a conclusion also supported by other independent studies (Moassefi et al., 2024; Marrone et al., 2019; Ligneris et al., 2023). Furthermore, the same problem was discovered in DL-EEG applications, where, from a review of 154 selected papers, only 12 were found to be easily reproducible (Roy et al., 2019).

The above statistics highlight not only the severity of the reproducibility crisis, but also how often this important issue is overlooked by both authors and publishers. Insufficient reproducibility not only threatens the credibility of scientific findings, potentially hindering the discovery of new knowledge in the domain (e.g., treatments for neurological disorders), but also introduces inconsistencies in results due to factors such as dataset diversity, variability in preprocessing, and discrepancies in model implementation and evaluation. Such inconsistencies heighten the risk of misinterpreting data or drawing incorrect conclusions that support the validity of a framework over another, thereby posing a potential negative impact on clinical outcomes. For instance, various studies using deep learning to classify different types of dementia or to predict cognitive scores with extremely high accuracies have been questioned not only for their lack of reproducibility, but also for potential performance biases arising from inadequate data partitioning methods or ambiguous validation or model selection procedures (Brookshire et al., 2024; Wen et al., 2020). In contrast, other disciplines have achieved greater reproducibility through the use of standardized datasets and methodologies. For example, the ImageNet dataset (Krizhevsky et al., 2012) has significantly advanced computer vision by establishing benchmarks categorized by learning methods, while the General Language Understanding Evaluation (GLUE) benchmark provides a collection of resources for training, evaluating, and analyzing natural language understanding systems (Wang et al., 2018).

Consequently, there is a need for the neuroimaging field to adopt similar practices to improve the reliability of published research, which heavily depends on its reproducibility. To contribute in this direction, this paper outlines the key elements necessary for achieving reproducibility in neuroimaging deep learning studies and organize them in a table that can be also used as a checklist.

## 2 Improving reproducibility

Reproducibility can only be achieved if considered since the design of the study. Even in such cases, deep learning presents numerous sources of irreproducibility, making it difficult for researchers to account for them all. Various checklists have been proposed to guide neuroscientists in designing DL studies (Roy et al., 2019; Moassefi et al., 2024), yet they often focus on specific data types or do not effectively explain how certain features affect reproducibility. To provide a clear checklist for researchers, this section introduces an updated table listing 30 key elements that are essential to ensuring reproducibility. Features in Table 1 are organized into six categories and discussed concisely in the following sub-paragraphs, highlighting how the absence of such information can hinder reproducibility and affect the reliability of results. Additionally, specific locations (paper, supplementary material, repository) are suggested to help authors improve clarity and reduce density in the papers.

**Table 1:** List of criteria a research study should fulfill to ensure reproducibility.

**Category**	**Feature**	**Placement**
**Software and hardware**		
1	Source code	Open repository (e.g., GitHub)
2	Software environment	Paper and source code
3	Computational resources	Paper
**Dataset**		
1	Raw or preprocessed data	Open platform (e.g., OpenNeuro)
2	Number of subjects	Paper and supplementary
3	Demographic data	If multiple datasets are used, provide a summary table within the paper's main body
4	Number of samples	
5	Acquisition modalities	
**Data preprocessing**		
1	Artifact handling algorithms	Paper, supplementary, source code
2	Normalization and standardization	Specify the parameters used for each preprocessing step and annotate manually performed operations
3	Harmonization and co-registration	
4	Resampling	
5	Data augmentation	
**Model**		
1	Architecture description	Paper, supplementary, source code
2	Number of learnable parameters	Provide a detailed summary table within the supplementary material
3	Input dimension	
**Training hyperparameters**		
1	Random seed	Paper
2	Parameter initialization	Provide all the information in a dedicated subsection
3	Batch size	
4	Number of epochs	Consider uploading the hyperparameter search results within the main repository, or within the supplementary material
5	Loss functions	
6	Optimization algorithms	
7	Learning rate and schedulers	
8	Stopping criteria	
9	Regularization	
10	Hyperparameters search method	
**Model evaluation**		
1	Data partition	Paper and supplementary
2	Validation scheme	Provide the result of each training within the main repository, or as a supplementary material
3	Performance metric	
4	Baseline comparison	

### 2.1 Software and hardware

Reproducibility is ensured only if authors share the source code within an open platform like GitHub.^1^ However, in neuroscience this is done only 10% of the times, in contrast with other domains like computer vision, where the percentage can reach 70% (Pineau et al., 2021). Sharing a well organized code is crucial, as it provides valuable insight into the implementation correctness. Additionally, releasing an easily executable code can facilitate the design of fair comparisons between the original and new results, which helps building useful benchmarks.

On the paper side, the name and version of each main software library should be clearly reported. This helps to limit any randomness introduced by unnoticed changes in the library's code base, which can potentially compromise the reproducibility and repeatability of results (Alahmari et al., 2020). For the same reason, the entire environment should be also exported and uploaded within the repository. Furthermore, authors should report additional computational details to help tracking other known hardware non-determinism (Chen et al., 2022). These include: GPU model and number, CUDA version, training time, memory allocation, and parallelization across devices.

### 2.2 Dataset

Previously cited review studies agree that only a small portion of researchers share the data on dedicated open platforms. Sharing health data is indeed problematic due to strict privacy regulations. However, researchers should not feel discouraged, since there are many tools nowadays designed to facilitate data de-identification, reorganization in standardized formats (e.g., BIDS Gorgolewski et al., 2016), and sharing with a digital object identifier (e.g., Zenodo,^2^ OpenNeuro^3^ Markiewicz et al., 2021). Sharing raw data not only enhances the reliability of research, but also encourages other teams to try improving the original results. In addition, increasing the number of public data can facilitate the creation of multi-center datasets, which are crucial for training DL models being able to better generalize on unseen data. For example, the increasing availability large open-source datasets has encouraged researchers to explore promising deep learning architectures, such as transformers, improving the investigation of the spatiotemporal dynamics of the human brain (Kim et al., 2023; Tang et al., 2023).

Describing the dataset in detail is also important. The paper should clearly indicate the number of subjects, relevant demographic data, number of training samples, and data acquisition modalities. Data acquisition modalities may include the channel map, sampling rate, and reference for EEG data, or the scanner model, voxel size, and acquisition sequence for MRI data.

### 2.3 Data preprocessing

Medical data are usually processed with complex pipelines, not necessary fully automatic. It is therefore extremely important to make this step reproducible, as different implementation of the same pipeline can lead to different results. For example, minimal changes in the IClabel's thresholds (Pion-Tonachini et al., 2019) can drastically alter the EEG's spectral properties; similarly, the convergence threshold in the ANTs software (Avants et al., 2014) can heavily affects the MRI registration. Consequently, any customizable parameter (or manually performed operation) should be clearly described in the paper. Alternatively, researchers may upload preprocessed data alongside the raw data, enabling consistent input for analysis and assisting laboratories with limited computational resources in reproducing results. Data augmentations should also be clearly described, with particular regard to the range of random values used during their execution.

### 2.4 Model architecture

Deep learning models should be described on several fronts, especially if architectures are particularly complex. First, the paper should include a schematic representation of the model, and specify its input dimension and number of trainable parameters. (If the model comes from an external library, its name and version should be reported.) Second, supplementary material should provide a full summary table where the number of parameters, input and output dimensions, and other customizable features (e.g., weight constraints, grouped convolutions) are listed for each layer of the network. Finally, the actual model's implementation should be uploaded within the source code, as different implementations are characterized by different parameters initialization.

### 2.5 Training hyperparameters

Table 1 lists a set of 10 training hyperparameters that should be described within the paper. For each of them, it is important to specify the value of each customizable parameter, if necessary in a table. Authors are encouraged to pay special attention to the use of random seeds as a way to control randomness in the code. Simply setting the random seed at the beginning of a script might not be enough. Best practice is to check if model's parameters after initialization and at the end of the training are the same on multiple rerun. Additionally, libraries like Pytorch (Paszke et al., 2019) can be configured to use a set of deterministic algorithms which, although slower, can enhance reproducibility in DL experiments.

### 2.6 Model evaluation

In neuroimaging data analysis, it is best practice to evaluate DL models with subject-based cross-validation procedures, which require to partition the dataset multiple times in training and validation sets (Kunjan et al., 2021). Partitions should be reproducible, because different splits can dramatically change the results due to strong subject-based characteristics that can guide the learning process toward different loss minima. Furthermore, it is important to clearly state how the validation set is used during training. While switching to a train-validation-test split scenario as part of a nested cross-validation, as proposed in Kim et al. (2022), is preferable, it is unfortunately usual to find works that use the validation set to stop the training. This process introduces data leakage and affects the reliability of the results. Nevertheless, this information is often omitted (Pandey and Seeja, 2022), leaving the question of whether data leakage was introduced unanswered.

## 3 Discussion

Designing a reproducible deep learning experiment, especially in a complex and heterogeneous field like neuroscience, is extremely challenging. Researchers must consider several factors that can influence experimental outcomes; otherwise, reproducibility can be permanently lost. In computational neuroscience, DL applications to neuroimaging data require multiple training instances to be run as part of subject-based cross-validation procedures (e.g., Leave-N-Subject-Out, typically using 5 or 10 folds Poldrack et al., 2020). Even with modern GPUs capable of performing 10^12^ floating point operations per second, these strategies can be time-consuming and computationally intensive. It is natural that researchers might hesitate to re-run the analysis if they encounter reproducibility issues. Additionally, deep learning advances rapidly, with numerous papers published every month. While this rapidity fuels the research teams' need to quickly disseminate their work, it should also not undermine reproducibility, which is central to the scientific method and a necessary (though not sufficient) condition for a scientific statement to be accepted as new knowledge (Colliot et al., 2023). Focusing on reproducibility indirectly compels researchers to develop methodologically robust experiments that yield more reliable results.

Technical reproducibility in DL studies can only be assured if both the code and dataset are released by the study group. Although privacy regulations can complicate data sharing, teams can nonetheless make their source code publicly available (Ciobanu-Caraus et al., 2024). This will provide a complementary resource that is often necessary for clearly describing neuroimaging deep learning studies, especially when articles are subjected to word or page limits. Table 1 not only lists 30 key reproducibility elements, but also suggests specific places to present them without hindering the paper's readability. However, this alone might not be enough. Actions are required also from the publisher side; in fact, many of them have begun revising their policies to strongly encourage, and occasionally mandate, the sharing of code and research data.

In conclusion, while deep learning is a powerful tool, it still remains extremely sensitive to minimal variations in the experimental setup. It is essential to re-evaluate priorities, especially in sensitive applications like medical ones, and look beyond mere model performance. Without addressing critical issues such as reproducibility and generalizability, reported accuracies may be seen as mere numbers lacking practical meaning.

## Footnotes

^1^ https://github.com

^2^ https://zenodo.org

^3^ https://openneuro.org
